# Role of Insulin Resistance in MAFLD

**DOI:** 10.3390/ijms22084156

**Published:** 2021-04-16

**Authors:** Yoshitaka Sakurai, Naoto Kubota, Toshimasa Yamauchi, Takashi Kadowaki

**Affiliations:** 1 Department of Diabetes and Metabolic Diseases, Graduate School of Medicine, The University of Tokyo, Tokyo 113-8655, Japan; sakuraichin-tky@umin.ac.jp (Y.S.); tyamau-tky@umin.net (T.Y.); 2 Department of Clinical Nutrition Therapy, The University of Tokyo, Tokyo 113-8655, Japan; 3 Clinical Nutrition Program, National Institutes of Biomedical Innovation, Health and Nutrition, Osaka 567-0085, Japan; 4 Toranomon Hospital, Tokyo 105-8470, Japan

**Keywords:** NAFLD, MAFLD, insulin signaling, insulin resistance, de novo lipogenesis (DNL), triglycerides (TG), diacylglycerol (DAG), lipid metabolism

## Abstract

Many studies have reported that metabolic dysfunction is closely involved in the complex mechanism underlying the development of non-alcoholic fatty liver disease (NAFLD), which has prompted a movement to consider renaming NAFLD as metabolic dysfunction-associated fatty liver disease (MAFLD). Metabolic dysfunction in this context encompasses obesity, type 2 diabetes mellitus, hypertension, dyslipidemia, and metabolic syndrome, with insulin resistance as the common underlying pathophysiology. Imbalance between energy intake and expenditure results in insulin resistance in various tissues and alteration of the gut microbiota, resulting in fat accumulation in the liver. The role of genetics has also been revealed in hepatic fat accumulation and fibrosis. In the process of fat accumulation in the liver, intracellular damage as well as hepatic insulin resistance further potentiates inflammation, fibrosis, and carcinogenesis. Increased lipogenic substrate supply from other tissues, hepatic zonation of Irs1, and other factors, including ER stress, play crucial roles in increased hepatic de novo lipogenesis in MAFLD with hepatic insulin resistance. Herein, we provide an overview of the factors contributing to and the role of systemic and local insulin resistance in the development and progression of MAFLD.

## 1. Introduction

The term non-alcoholic fatty liver disease (NAFLD) was coined by Ludwig in 1980 to describe fatty liver disease of unknown cause in subjects without a history of excessive alcohol intake [1]. NAFLD has been categorized histologically into non-alcoholic fatty liver (NAFL) or non-alcoholic steatohepatitis (NASH); the former is defined as the presence of ≥5% hepatic steatosis without evidence of hepatocellular injury in the form of hepatocyte ballooning, while the latter is defined as the presence of ≥5% hepatic steatosis, associated with inflammation and hepatocyte injury (e.g., hepatocyte ballooning), with or without fibrosis [2]. The global prevalence of NAFLD is estimated at 24%; the highest rates are reported from South America and the Middle East, followed by Asia, the USA, and Europe [3]. Whereas it has been known that the vast majority of patients diagnosed as having isolated fatty liver (NAFL) on initial biopsy are at a low risk for the development of more advanced liver disease [4], a recent prospective cohort study with a mean follow-up time of 19.8 years showed that 16% and 9% of patients with NAFLD developed advanced fibrosis and end-stage liver disease, respectively [5], suggesting that the prognosis of NAFLD cannot be underestimated at all. Although NASH with cirrhosis is thought to predispose to hepatocellular carcinoma (HCC), hepatocarcinogenesis has also come to be increasingly recognized in NASH patients without cirrhosis [6]. In regard to the apparently complex mechanism involved in the progression from NAFLD to NASH/HCC, inflammatory mediators derived from various tissues could play a central role in the cascade of inflammation, fibrosis, and finally tumor development [7], and metabolic dysfunction is considered to be closely associated with these kinds of inflammatory changes.

It has been reported that patients with NAFLD are among those at the greatest risk for the development of type 2 diabetes mellitus (T2DM). A systematic review and meta-analysis showed that NAFLD (diagnosed based on either elevation of the serum levels of liver enzymes or the findings of ultrasonography) is associated with an approximately twofold increased risk of incident T2DM and metabolic syndrome over a median period of 5 years [8]. Another meta-analysis reported that metabolic comorbidities were frequently associated with NAFLD, including obesity (51.34%), T2DM (22.51%), dyslipidemia (69.16%), hypertension (39.34%), and metabolic syndrome (42.54%) [9].

Another study showed that almost all patients with T2DM have NASH, regardless of the liver enzyme levels, suggesting that steatohepatitis may represent the sole feature of liver damage in patients with type 2 diabetes mellitus [10]. Besides metabolic dysfunction, NAFLD may also coexist with other liver diseases. The prevalence of NAFLD is similar between groups of individuals with and without suspected liver disease (elevated serum levels of liver enzymes, hepatitis B surface antigen, or hepatitis C virus RNA positivity) [11]. Whereas the impact of hepatitis B virus (HBV) infection on hepatic steatosis still remains controversial, chronic liver infection with hepatitis C virus (HCV) is known to be directly associated with hepatic steatosis, suggesting that coexistence of chronic HCV hepatitis and metabolic comorbidities could increase the risk of development of NAFLD [12]. Since the common pathophysiological abnormality underlying obesity, T2DM, and metabolic syndrome is insulin resistance, NAFLD is regarded as one of the phenotypes of the pathophysiological conditions associated with insulin resistance.

Certain medications may also adversely affect the risk of progression of NAFLD through exacerbating insulin resistance. While altered morphine metabolism and distribution has been reported in patients with liver disease [13], opioid use is more common in NAFLD patients with cirrhosis [14] as well as other liver diseases [15]. Since opioid peptides have been reported to play roles in some metabolic events associated with the development of obesity [16], use of some drugs such as opioids might be associated with development of NAFLD in patients with other liver diseases, including viral hepatitis and autoimmune diseases.

In addition to reports of the increased incidence of cardiovascular diseases (CVDs) in insulin-resistant patients with pre-diabetes or normal glucose tolerance [17,18], NAFLD itself has also been reported to be strongly associated with an elevated risk of CVD [19]. Moreover, the coexistent NAFLD and T2DM is known to be associated with an increased risk of development of chronic macrovascular and microvascular complications via several mechanisms, such as increased insulin resistance, dyslipidemia, upregulated proinflammatory cytokines, dysbiosis, and alterations of hemostatic–fibrinolytic factors [20]. Based on the above, NAFLD is still considered as being the hepatic component of metabolic syndrome from the perspective of the risk of CVD.

## 2. Renaming NAFLD to MAFLD

The current definition of NAFLD requires the exclusion of other causes of liver diseases and of a history of excessive alcohol intake. However, the precise definition of excessive alcohol consumption in patients with suspected NAFLD is uncertain. A consensus meeting recommended that for the purposes of eligibility for NASH clinical trials, significant alcohol consumption be defined as >21 standard drinks per week for men and >14 standard drinks per week for women over a 2-year period preceding the baseline liver histology [2]. Moderate alcohol consumption has also been demonstrated to be significantly and independently associated with worsening of hepatic fibrosis, suggesting that even moderate alcohol consumption might be harmful [21]. In the clinical setting, it seems difficult to grasp the exact amount of drinking by patients. Based on the fact that NAFLD could coexist with other liver diseases, including viral hepatitis and autoimmune diseases, the expression of “non-alcoholic” does not appear to accurately reflect the pathogenesis of this heterogeneous liver disease. Recently, experts reached the consensus that “metabolic dysfunction-associated fatty liver disease or MAFLD” may be a more appropriate and inclusive definition [22]. The criterion for the diagnosis of MAFLD would be: evidence of hepatic steatosis (detected by imaging, blood biomarkers, or liver histology), associated with any one or more of the following: overweight/obesity, T2DM, evidence of metabolic dysregulation [23].

## 3. Factors Underlying the Pathogenesis of MAFLD

Various factors are recognized to be involved in the development and progression of MAFLD. Several gene variants are reported to serve as genetic susceptibility genes for MAFLD. In addition, pathological alterations of the gut microbiota and increased insulin resistance in the adipose tissue and skeletal muscle are known to affect hepatic lipid metabolism, promoting further hepatic fat accumulation and inflammation.

### 3.1. Genetic Background

**Figure 1:**
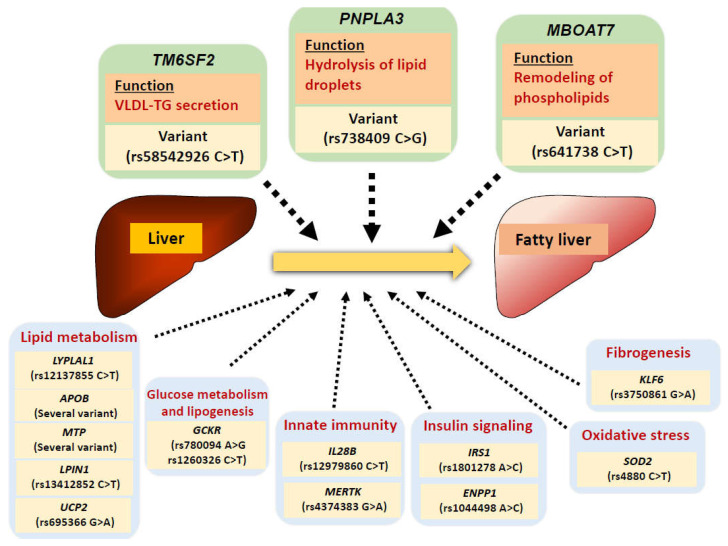
Summary of genetic variants related to the pathogenesis of NAFLD. Many genome-wide association studies have identified novel loci associated with disease severity phenotypes of NAFLD. The *PNPLA3* (rs738409 C>G) variant, which causes impaired mobilization of TG from lipid droplets, predisposes to progression along the full spectrum of liver damage associated with fatty liver. The *TM6SF2* (rs58542926 C>T) variant, a loss-of-function variant, is associated with hepatic steatosis via decreased VLDL-TG secretion in the liver. The *MBOAT7* (rs641738 C>T) variant, resulting in lower MBOAT7 protein expression in the liver, is associated with increased hepatic fat content through inducing changes in the remodeling of phospholipids. In addition, other genetic variants known to be involved in the regulation of lipid metabolism (*LYPLAL1*, *APOB*, *MTP*, *LPIN1*, *UCP2*), glucose metabolism and lipogenesis (*GCKR*), innate immunity (*IL28B*, *MERTK*), insulin signaling (*ENPP1*, *IRS1*), oxidative stress (*SOD2*), and fibrogenesis (*KLF6*) have also been reported to be associated with the progression of NAFLD.

Many genome-wide association studies have identified novel loci associated with disease severity phenotypes of NAFLD and NASH [24,25] (Figure 1). While PNPLA3 (patatin-like phospholipase domain-containing protein 3), which hydrolyzes glycerolipids, is known to be expressed in a variety of tissues, particularly high expression levels have been reported in the liver and kidneys [26]. Romeo et al. identified an allele in the *PNPLA3* (rs738409; I148M) gene that was strongly associated with increased hepatic fat levels and hepatic inflammation [27]. Several studies have confirmed that the I148M variant predisposes to progression along the full spectrum of liver damage associated with fatty liver, from simple steatosis to steatohepatitis and progressive fibrosis, and could promote fibrogenesis in other liver diseases, including alcoholic liver diseases, viral hepatitis, and autoimmune liver diseases [28]. The *PNPLA3* rs738409 C > G polymorphism has also been reported to be associated with an elevated risk of development of HCC [29]. The I148M substitution was reported to cause loss-of-function of PNPLA3 [30]. Using knock-in mice of PNPLA3-I148M, it was demonstrated that this variant disrupts ubiquitylation and proteasomal degradation of PNPLA3, resulting in the accumulation of PNPLA3-I148M and impaired mobilization of TG from lipid droplets [31].

TM6SF2, reported to be expressed mainly in the intestine and liver, seems to be related to VLDL-TG secretion in the liver [26]. The TM6SF2-E167K variant has also been identified as a loss-of-function variant associated with hepatic steatosis. Knockdown of *Tm6sf2* in mice increased the liver TG content and decreased VLDL secretion [32], suggesting that TM6SF2 is related to hepatic secretion of TG. While subjects with the TM6SF2-E167K variant are more susceptible to progressive NASH, the incidence of cardiovascular events in these patients is reported to be lower, because of the lower levels of circulating lipids [33].

Variants of the MBOAT7 gene, which has been reported to be the risk locus for alcohol-related cirrhosis [34], have also been shown to be associated with the development and severity of NAFLD [35]. The rs641738 polymorphism of *MBOAT7* is associated with increased hepatic fat content, lower MBOAT7 protein expression in the liver, and changes in the plasma phosphatidylinositol species [36]. Mammalian membrane-bound O-acyltransferase 7 (MBOAT7), also known as lysophosphatidylinositol acyltransferase 1, plays a major role in regulating arachidonic acid availability for eicosanoid biosynthesis [37]. It was reported that mice with hepatocyte-specific deletion of MBOAT7 showed spontaneous steatosis with increased hepatic cholesterol ester content, hepatic fibrosis with minimal inflammation after receiving a high-fat, low-methionine, choline-deficient diet, and increased plasma total lysophosphatidylinositol levels, suggesting that Mboat7 deficiency may trigger an inflammation-independent pathway of liver fibrosis [38]. Recently, another group reported that direct metabolic flow from phosphatidylinositol to TG caused by hepatic MBOAT7 depletion resulted in an increase in the total hepatic TG content [39].

Besides the above, variations of the glucokinase regulator (*GCKR*) gene locus have been reported to be associated with NAFLD, and various studies have demonstrated the roles of other genetic variations in the regulation of lipid metabolism (*LYPLAL1*, *APOB*, *MTP*, *LPIN1*, *UCP2*), innate immunity (*IL28B*, *MERTK*), insulin signaling (*ENPP1*, *IRS1*), oxidative stress (*SOD2*), and fibrogenesis (*KLF6*) in relation to the progression of NAFLD [24] (Figure 1).

### 3.2. Alterations of Lipid Metabolism in the Liver

#### 3.2.1. Increase in De Novo Lipogenesis

Liver triglyceride (TG) is synthesized from fatty acyl CoA. The hepatocellular concentration of fatty acyl CoA is determined from the balance between free fatty acid (FFA) formation (from circulating FFAs, de novo lipogenesis (DNL), lipoprotein uptake, and TG breakdown) and utilization (lipid synthesis and oxidation) [40]. In an experiment conducted with isotopes, a threefold increase of DNL and higher nocturnal plasma levels of FFAs were observed in patients with NAFLD [41]. In NAFLD and obese patients, the TG composition of the liver is reported to be 59% from NEFA, 26% from DNL, and 15% from the diet, suggesting that DNL is elevated in the fasting state in NAFLD and that the NEFA pool is the primary contributor to hepatic TG in NAFLD [42]. There are two major transcriptional factors for DNL: sterol regulatory element binding protein 1c (SREBP1c), regulated by insulin signaling, and carbohydrate response element binding protein (ChREBP), activated by glucose uptake [43]. Among the peroxisome proliferator-activated receptors (PPARs), PPARγ appears to upregulate the expressions of genes involved in lipogenesis (such as *Acc1*, *Cd36*, and *Scd1*), as well as triglyceride synthesis (*Mogat1*) and lipid droplet formation (*Fsp27*, *Plin2*, and *Cidea*) [44]. Although upregulation of hepatic PPARγ has been observed in mice with hepatic steatosis, previous studies using genetically modified mouse models identified the different roles of PPARγ in hepatocytes and non-liver parenchymal cells, indicating the complicated mechanism of hepatic lipogenesis mediated by PPARγ in the liver [45]. Moreover, PPARγ expression has been reported not to be correlated with the severity of NASH in humans [46]. The precise role of PPARγ in the development of MAFLD is not yet known.

#### 3.2.2. Increased Mitochondrial Fatty Acid Oxidation

In response to lipid overload, oxidative and TCA cycle activities are increased, which potentiates oxidative stress and liver damage during chronic hepatic steatosis [47]. Koliaki et al., proposed that the “hepatic mitochondrial flexibility” could be a compensatory and protective response in the early stages of NAFLD. In their study, the maximal respiration rates of isolated mitochondria were higher in obese persons with or without NAFL than in lean persons in spite of similar mitochondrial contents, which was abolished in obese patients with NASH. Elevated hepatic insulin resistance, mitochondrial uncoupling, and leaking activity might result in augmented hepatic oxidative stress and inflammatory response [48]. Indeed, increased lipid delivery caused by either intralipid infusion or a high-fat diet (HFD) not only induced oxidative metabolism, but also caused a proportional increase in oxidative stress and inflammation [49]. Thus, the state of fatty acid oxidation in NAFLD seems to depend on the progression of the disease spectrum, and relatively impaired mitochondrial oxidative capacity is associated with hepatocyte damage through enhanced oxidative stress and inflammation. As many studies imply an important role of mitochondria in the pathogenesis of metabolic disorders such as obesity, metabolic syndrome, and T2DM [50], mitochondrial dysfunction in the liver is also considered to be a significant contributor to the pathogenesis of MAFLD.

#### 3.2.3. Increased Uptake of FFAs

In regard to FFA uptake by the liver, many studies have shown that CD36, which facilitates intracellular FFA uptake and trafficking, might contribute to the progression to NASH [51]. In a mouse model, hepatocyte-specific CD36 overexpression was associated with increased hepatic TG storage and secretion [52]. In contrast, hepatocyte-specific disruption of CD36 attenuated fatty liver induced by an HFD and in a genetic model with increased circulating FFAs [53]. The expression of CD36 was found to be upregulated in patients with high hepatic fat content [54]. Hepatic CD36 upregulation was also significantly associated with insulin resistance, hyperinsulinemia, and increased steatosis in patients with NASH [55]. CD36 localization on the plasma membranes of hepatocytes was markedly increased in patients with NASH as compared to people with normal livers or simple hepatic steatosis, in which CD36 palmitoylation plays a key role via increased CD36 protein hydrophobicity [56].

#### 3.2.4. Alterations in TG Secretion

**Figure 2:**
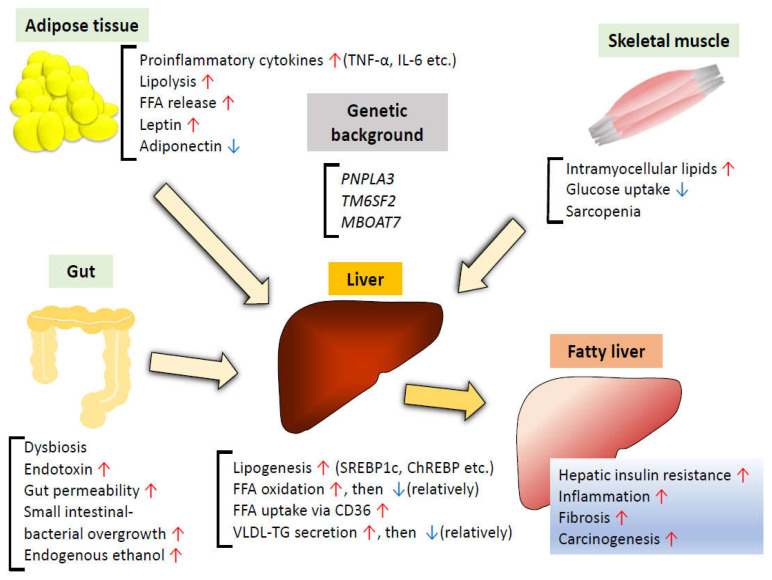
Summary of factors involved in the pathogenesis of MAFLD. Imbalance between energy intake and expenditure leads to fat accumulation in adipose tissue. When the accumulation exceeds the capacity for adipose tissue remodeling, increased release of FFAs and proinflammatory cytokines with dysregulated production of adipokines results in the development of insulin resistance in the skeletal muscle as well as adipose tissue. Glucose and FFAs not utilized in the skeletal muscle and adipose tissue become substrates for hepatic DNL. Increased uptake of FFAs and relatively decreased VLDL-TG secretion also promote hepatic fat accumulation. Compensatory increase in FFA oxidation is accompanied by enhanced oxidative stress and inflammation. Dysbiosis, defined as an imbalance in the microbiota composition, can disrupt the tight junctions in intestinal endothelial cells, leading to increased mucosal permeability and translocation of bacterial LPS from bacteria. Small intestinal bacterial overgrowth and gut-derived metabolites, such as endogenous ethanol, are also associated with the progression of MAFLD. In addition, people with specific genetic risk variants are also susceptible to the development and progression of MAFLD. Taken together, insulin resistance in adipose tissue and the skeletal muscle, dysbiosis, and genetic predisposition are synergistically involved in fat accumulation and inflammation in the liver, followed by subsequent fibrosis and carcinogenesis, in patients with MAFLD.

As mentioned above in relation to *TM6SF2*, TG secretion is also involved in NAFLD progression. Obese patients with NAFLD show increased VLDL-TG secretion, although the increase in hepatic TG export is not sufficient to normalize the intrahepatic TG content [57]. In patients with NAFLD in which insulin-induced endogenous glucose production was suppressed, impaired insulin-induced suppression of VLDL-TG secretion was observed [58], suggesting that the increase in VLDL-TG secretion may be an early pathophysiological manifestation of NAFLD. Apolipoprotein B100 (ApoB100) and microsomal TG transfer protein (MTP) are required for the formation of mature VLDL particles. Hepatic steatosis, secondary to compromised TG export, is common in patients with genetic defects in the *ApoB* or *MTP* gene [59]. Whereas ApoB100 synthesis is a rate-determining step in hepatocyte lipid export, decreased synthesis of this protein was observed in patients with NASH [60], suggesting that impaired VLDL-TG secretion exacerbates NAFLD. Hepatic induction of *MTP* in a mouse model of hereditary fatty liver without obesity led to a reduction in hepatic TG accumulation, improvement of VLDL export, amelioration of NASH-like lesions, as well as glucose intolerance [61]. Despite a greater degree of impairment of VLDL synthesis and secretion in cases of NASH than in those of NAFL, the hepatic TG content was found to be similar between NAFL and NASH, suggesting that impairment of VLDL synthesis might result in increased lipid oxidation and DNA oxidative damage, resulting in the progression to NASH [62] (Figure 2).

### 3.3. Insulin Resistance in Adipose Tissue

During the development of obesity, various changes are observed in the microenvironment of the adipose tissue. While under physiological conditions, adipose tissue expands through effective recruitment of adipogenic precursor cells along with an adequate angiogenic response and appropriate remodeling of the extracellular matrix, pathological adipose tissue expansion consists of massive enlargement of existing adipocytes, limited angiogenesis, and hypoxia, resulting in the activation of HIF-1α, induction of a fibrotic program, and systemic insulin resistance, accompanied by activation of proinflammatory macrophages [63]. As insulin inhibits lipolysis in the adipocytes [64], insulin resistance in the adipose tissue causes increased release of FFAs. Mice with adipose tissue-specific knockout of the insulin receptor exhibited lipodystrophy and severe HFD-induced fatty liver [65]. Humans with congenital generalized lipodystrophy develop extreme insulin resistance and its complications, such as T2DM, hyperlipidemia, and fatty liver [66]. Indeed, in humans, visceral adipose tissue lipolysis accounts for an increasing proportion of hepatic FFA delivery as the visceral fat increases [67]. NAFLD patients have significantly higher serum FFA levels, and serum FFA level has been identified as an independent predictor of advanced fibrosis in NAFLD patients [68]. Circulating FFA increases activation of the proinflammatory nuclear factor-kappa B (NF-kappa B) pathway in the liver [69]. Thus, the release of fatty acids from dysfunctional and insulin-resistant adipocytes results in lipotoxicity, leading to the accumulation of triglyceride-derived toxic metabolites in ectopic tissues, including the liver [70].

Activation of proinflammatory macrophages during pathological adipose tissue expansion leads to upregulation of the proinflammatory cytokine tumor necrosis factor-α (TNF-α), which not only promotes adipocyte lipolysis [71] but also inhibits insulin-stimulated tyrosine phosphorylation of insulin receptor substrate1 (Irs1) by enhancing phosphorylation of Ser307 on Irs1 [72]. These effects were mediated by the activated IKKβ-NF-κB pathway [73]. TNF-α is also known to play an important role in the development of systemic insulin resistance [74], and overexpression of TNF-α mRNA has been reported in the liver and adipose tissue of NASH patients [75], suggesting that upregulated TNF-α in the adipose tissue might be involved in the progression of NAFLD via increasing systemic insulin resistance, as well as promoting inflammation in various tissues. Enhanced secretion of interleukin-6 (IL-6) from the visceral adipose tissue has been observed in obese individuals [76]. JNK1-dependent secretion of IL-6 in the adipose tissue caused increased expression of hepatic SOCS3, which induced degradation of molecules involved in the insulin signaling, resulting in hepatic insulin resistance [77]. Serum IL-6 levels are reported to be significantly increased in NAFLD and NASH patients as compared to healthy individuals [78]. Although IL-6 exerts both pro- and anti-inflammatory activities depending on the context [79], chronic low-grade inflammation mediated by IL-6 in the adipose tissue could be related to the progression of NAFLD.

Besides the adipocytes and the stromal vascular fraction secreting various proinflammatory cytokines, adipocytes also secrete more specialized adipokines, such as leptin and adiponectin [80]. Leptin is known to be associated with food intake, energy homeostasis, metabolism, cognition, immune functions, and bone metabolism [81]. Although circulating leptin levels are directly proportional to the amount of body fat, leptin resistance is also seen in obese people with decreased leptin-induced suppression of appetite and weight gain [81]. Circulating leptin levels have been reported to be higher in patients with NAFLD than in controls, and higher levels of circulating leptin have been shown with increasing severity of NAFLD [82]. In addition to leptin promoting profibrogenic responses via TGF-β signaling [83,84], obesity-induced leptin exacerbates NASH via enhancing the responsiveness to endotoxins [85], suggesting that leptin plays a key role in proinflammatory, profibrogenic processes involved in the progression of NAFLD. On the other hand, adiponectin has the effect of ameliorating NAFLD. Adiponectin has two major receptors, AdipR1 and AdipoR2. In the liver, AdipoR1 is involved in the activation of AMP-activated kinase (AMPK), and AdipoR2 is involved in the activation of PPARα, both of which lead to increased insulin sensitivity [86]. As obesity is associated with reduced adiponectin levels via multiple mechanisms, including decreased levels of SirT1, hyperinsulinemia, oxidative stress, and inflammation [87], decreased levels of circulating adiponectin in NAFLD are related to a decrease in hepatic insulin sensitivity and increase in the amount of hepatic fat content [88]. Administration of recombinant adiponectin to either mice fed high-fat ethanol-containing food or non-alcoholic obese ob/ob mice alleviated steatosis and attenuated inflammation [89]. Recently, it was reported that treatment with a dual AdipoR1/AdipoR2 agonist improved both NASH and fibrosis in mice models via reduced hepatic stellate cell activation [90] (Figure 2). While several observational studies have reported elevated serum levels of other obesity-related adipokines, such as resistin, visfatin, and RBP4 in NAFLD patients, some contradictory results have also been reported, suggesting that the causal relationship between these adipokines and NAFLD remains under debate [91,92].

### 3.4. Insulin Resistance in the Skeletal Muscle

Skeletal muscle plays a major role in the regulation of blood glucose via insulin-stimulated glucose transport into the skeletal muscle mediated by GLUT4 [93]. The glucose taken up is used for glycogen synthesis and glycolysis. Many studies have indicated that skeletal muscle insulin resistance is the primary defect in T2DM [94]. Excess fatty acid supply promotes skeletal muscle insulin resistance together with an increase in intramyocellular lipids, leading to impaired insulin signaling induced by lipid-derived metabolites, such as diacylglycerols (DAGs) [70]. The muscle-specific insulin receptor-knockout (MIRKO) mice were normoglycemic, but exhibited elevated fat mass, and elevated serum levels of TG and FFA [95]. In MIRKO mice, insulin-stimulated muscle glucose transport and glycogen synthesis were decreased, whereas insulin-stimulated glucose transport in the adipose tissue was increased, suggesting that selective inactivation of muscle insulin signaling resulted in redistribution of substrates from the muscle to adipose tissue [96]. Glucose not utilized in the skeletal muscle could also serve as a substrate for hepatic DNL. In humans, insulin-resistant subjects exhibited reduced muscle glycogen synthesis and increased hepatic DNL [97]. A comparison of healthy, normal-weight, sedentary elderly subjects with younger subjects revealed that hepatic DNL occurred at a more than twofold higher level in the elderly than in the younger subjects, and that the postprandial hepatic TG content was approximately threefold higher in the former than in the latter group [98]. Conversely, improved muscle insulin responsiveness with exercise resulted in an increase in postprandial net muscle glycogen synthesis, leading to reduced hepatic TG synthesis, accompanied by a decrease in hepatic DNL [99]. Using the specific index of skeletal muscle insulin sensitivity (Matsuda index), which is calculated from the plasma glucose and insulin concentrations in the fasting state and during the OGTT [100], it was found that liver steatosis was associated with insulin resistance in the skeletal muscle rather than in the liver in patients with NAFLD [101]. In relation to skeletal muscle insulin resistance, sarcopenia, defined as reduced muscle quantity or quality, is a progressive and generalized skeletal muscle disorder that is associated with increased likelihood of certain adverse outcomes, such as falls, fractures, physical disability, and mortality [102]. In a prospective observational cohort study, individuals with a low muscle mass were found to be at an increased risk of development of NAFLD, even after adjusting for confounding factors, including insulin resistance and inflammation [103]. In a cohort of biopsy-proven NAFLD, sarcopenia was significantly associated with NASH and significant fibrosis, independent of obesity, inflammation, and insulin resistance [104]. As there seems to be an overlap in the pathophysiology of NAFLD and sarcopenia, it remains unclear if sarcopenia is the cause or consequence of progression of NAFLD [105] (Figure 2).

### 3.5. Gut Microbiota

Changes in the gut microbiota have been shown to be associated with metabolic syndrome, characterized by chronic low-grade inflammation, obesity, hyperglycemia, and dyslipidemia, as well as with intestinal diseases and neurological diseases [106]. Many studies have provided evidence to suggest that changes in the gut microbiota contribute to the pathogenesis of NAFLD via various mechanisms (Figure 2).

#### 3.5.1. Dysbiosis

Dysbiosis is defined as an imbalance in the microbiota composition. Since the microbiota respond rapidly to large changes in the diet, diet is thought to be as an important modulator of the gut microbiota [107]. There are several reports of associations between the severity of NAFLD and qualitative changes in the intestinal bacterial flora [108,109,110,111]. Although findings in relation to specific changes in the microbiota associated with the progression of NAFLD are not consistent, due to factors such as differences in the diets and ethnicities of the study populations, dysbiosis of the gut microbiota is considered to be involved in the development of NAFLD.

#### 3.5.2. Increased Gut Permeability

Dysbiosis can disrupt the tight junctions in the intestinal endothelial cells, leading to increased mucosal permeability and translocation of bacterial LPS from Gram-negative bacteria [112]. Actually, patients with NAFLD had significantly increased gut permeability, which was correlated with the severity of steatosis, but not with the presence of NASH [113]. Thus, dysbiosis is frequently associated with increased production of endotoxins from bacteria. In addition to the increase in the proportion of an LPS-containing microbiota in the gut following HFD feeding, mice with endotoxemia induced by continuous subcutaneous infusion of LPS showed exacerbation of glucose intolerance, accompanied by increased adipose tissue inflammation and hepatic TG content [114]. An increase in the plasma endotoxin activity was also observed after a Western-style diet for 1 month in healthy human subjects [115]. The endotoxin levels were significantly increased in NAFLD patients, with marked elevation noted in the early stages of fibrosis [116]. Activation of Toll-like receptors (TLRs) by bacterial LPS, especially TLR-4, in the Kupffer cells leads to a proinflammatory response, resulting in hepatic fibrogenesis [117]. As described above, increased leptin in people with obesity enhanced responsiveness to endotoxin, and activated STAT3 signaling through CD14, which is co-receptor of TLR4 [85].

#### 3.5.3. Small Intestinal Bacterial Overgrowth (SIBO)

In previous experiments using animal models, small intestinal bacterial overgrowth (SIBO), characterized by excessive bacteria in the small intestine, was shown to induce hepatic injury [118]. The prevalence of SIBO is reported to be higher in morbidly obese patients than in healthy subjects [119]. A recent meta-analysis confirmed a significant association between NAFLD and SIBO [120]. SIBO has been reported to be associated with systemic endotoxemia in patients with cirrhosis [121], and NAFLD patients with SIBO have also been demonstrated to exhibit significantly higher serum endotoxin levels [122]; thus, SIBO may affect the pathogenesis of NAFLD through causing endotoxemia.

#### 3.5.4. Gut-Derived Metabolites

The increased abundance of alcohol-producing bacteria in NASH microbiomes has been reported to promote liver inflammation [123]. Indeed, higher breath ethanol concentrations have been reported in obese individuals as compared to leaner individuals, among both mice [124] and human [125], independent of ethanol ingestion. Since ethanol is known to be associated with the increased gut permeability and endotoxemia in alcohol-induced liver disease [126,127], it is speculated that endogenous ethanol production by some kinds of gut microbiota might contribute to the pathogenesis of NAFLD. In addition, increased choline metabolism by the gut microbiota, altered bile acid metabolism, and short chain fatty acids (SCFAs) (acetate, propionate, and butyrate) formed during bacterial fermentation might also affect the pathogenesis of NAFLD, although the precise underlying mechanisms are not yet fully understood [128].

## 4. Role of Insulin Signaling in Hepatic De Novo Lipogenesis

**Figure 3:**
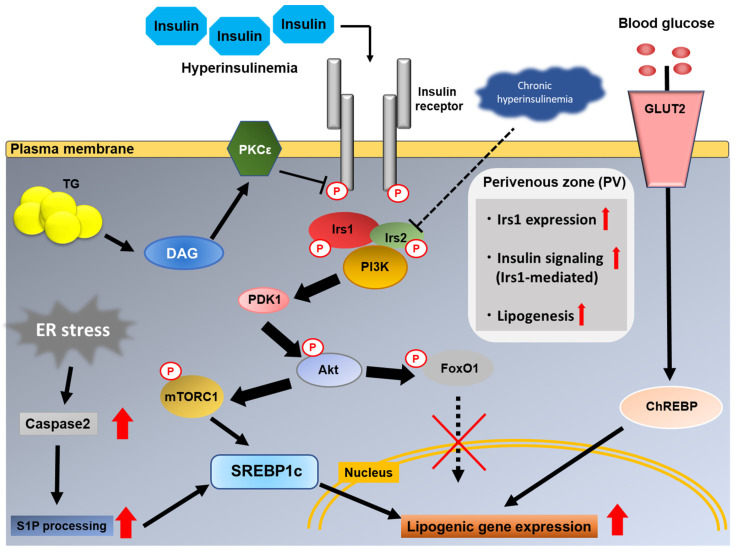
After insulin binds to the insulin receptor, autophosphorylation of tyrosine residues in the intracellular subunit of each of the receptors causes docking and phosphorylation of Irs1/Irs2, followed by activation of the downstream kinase cascades, such as the PI3K/Akt pathway. For induction of SREBP1c, mTORC1 activation and concomitant inhibition of FoxO1 via the PI3K/Akt pathway are required. In addition to the DAG-PKCε pathway inhibiting physiological phosphorylation of the insulin receptor, hyperinsulinemia leads to downregulation of hepatic Irs2 expression, resulting in the progression of hepatic insulin resistance. GLUT2 is the major glucose transporter in hepatocytes. In response to an increase in the intracellular glucose concentration, ChREBP induces expression of various genes related to lipogenic enzymes, independent of insulin signaling. Due to elevated Irs1 expression in the perivenous (PV) zone, which is the primary site of lipogenesis, insulin signaling is rather enhanced in the presence of hyperinsulinemia despite the downregulation of Irs2, resulting in increased lipogenesis and development of steatosis. Independent of insulin signaling, ER stress also activates SREBP through increased cleavage of S1P by caspase-2.

After insulin binds to the insulin receptor, autophosphorylation of tyrosine residues in the intracellular subunit of each of the receptors causes docking and phosphorylation of the insulin receptor substrates, followed by activation of downstream kinase cascades, such as the phosphatidylinositol-3 kinase (PI3K)/Akt pathway [129]. Activated AKT phosphorylates FoxO1, which subsequently reduces the transcriptional activity of various genes, including those encoding G6Pase and PEPCK, that are involved in gluconeogenesis. While activated Akt suppresses hepatic gluconeogenesis, it mediates hepatic lipid metabolism via activation of SREBP1c. Sterol-regulatory element binding protein-1c (SREBP1c) is regulated at both the transcriptional and proteolytic processing levels by mTORC1, one of the downstream molecules in the Akt signaling pathway [130] (Figure 3). SREBP cleavage-activating protein (SCAP) is a polytopic membrane protein that forms a complex with SREBPs in the ER and transports them to the Golgi apparatus for cleavage. After the NH2-terminal domain of SREBP reaches the nucleus and acts as a transcription factor, activated SREBP1c upregulates various lipogenic genes, including genes encoding acetyl-CoA carboxylase (ACC), fatty acid synthase (FAS), ATP citrate lyase, and genes encoding the enzymes of the fatty acid elongase complex, leading to activation of the fatty acid biosynthetic pathway [131]. Nuclear SREBP1c protein levels were significantly elevated in the livers of ob/ob and aP2-SREBP1c mice [132].

SCAP-deficient mice show a reduction in the basal rates of cholesterol and fatty acid synthesis in the liver [133]. Liver-specific deletion of SCAP in ob/ob mice also prevented fatty liver despite persistent obesity, hyperinsulinemia, and hyperglycemia [134], suggesting that SREBP1c plays a key role on hepatic DNL. A number of studies have attempted to identify the lipogenic roles of molecules involved in hepatic insulin signaling using genetically modified mouse models of insulin signaling (Table 1).

### 4.1. Insulin Receptor

To directly evaluate the metabolic effect of the insulin signaling in the liver, liver-specific insulin receptor knockout (LIRKO) mice were generated [135]. These mice exhibited marked insulin resistance, severe glucose intolerance, impaired glycogen storage, and a failure of insulin-induced suppression of hepatic glucose production. The metabolic phenotype became less severe with age-dependent progression of liver dysfunction. Impaired glucose tolerance, impaired suppression of hepatic glucose production, and failure of induction of lipogenic genes were reproduced in inducible insulin receptor-knockout mice [136]. In addition, LIRKO mice also showed high circulating levels of the leptin receptor, with normal leptin sensitivity, suggesting that insulin signaling in the liver also plays an important role in the control of leptin receptor expression and shedding [137]. Another group reported that LIRKO mice showed a decrease in the plasma levels of insulin-like growth factor 1, delayed growth during the first 2 months of life, and derangements in fatty acid metabolism, with decreased expression levels of the lipogenic enzymes, hepatic DAG, and plasma TG [138]. In regard to the lipoprotein profile of the LIRKO mice, these mice exhibited reduced plasma levels of HDL cholesterol and increased plasma levels of cholesterol-enriched ApoB containing lipoprotein particles, coupled with decreased TG secretion, resulting in an elevated risk of atherosclerosis [139].

While insulin-independent induction of SREBP1c through mTORC1 can occur after feeding, induction of lipogenic gene expressions in ob/ob mice or HFD-fed mice requires insulin signaling. Indeed, LIRKO mice, as well as inducible LIRKO mice generated with antisense oligonucleotides, showed reduction in the nuclear SREBP1 levels, lipogenic gene expressions, and hepatic TG content [140]. Although additional deletion of FoxO1 in the LIRKO mice rescued glucose tolerance and allowed for normal suppression of gluconeogenic gene expression after feeding or systemic administration of insulin, induction of lipogenic gene expressions by feeding was still suppressed [136,138], suggesting that hepatic insulin signaling other than via the IR-Akt-FoxO1 pathway is required for SREBP1c induction and DNL in vivo.

### 4.2. Insulin Receptor Substrate

Stimulation of insulin receptors with increased tyrosine kinase activity leads to autophosphorylation and phosphorylation of receptor substrates (Irs). Like the LIRKO mice, liver-specific Irs1/Irs2 double-knockout (LIrs1/2DKO) mice also exhibited impaired glucose tolerance with hyperinsulinemia and impaired suppression of hepatic glucose production [141,142]. Liver-specific Irs1-knockout mice (LIrs1KO) failed to exhibit insulin resistance during fasting, but showed insulin resistance after refeeding; conversely, liver-specific Irs2-knockout (LIrs2KO) mice exhibited insulin resistance during fasting, but not after refeeding. In relation to the suppression of SERBP1c by insulin signaling, the expression levels of SREBP1c in LIrs1KO mice were significantly lower only after refeeding, whereas in the LIrs2KO mice, by contrast, the expression levels of SREBP1c were significantly reduced only in the fasting state [141]. While all of the LIrs1KO, LIrs2KO, and LIrs1/2DKO mice exhibited insulin resistance and glucose intolerance under an HFD diet, only the LIrs2KO mice, and not the LIrs1KO or LIrs1/2DKO mice, showed hepatic steatosis [143].

### 4.3. PI3K

Class I phosphatidylinositol 3-kinase (PI3K), consisting of the p85 regulatory subunit and p110 catalytic subunit, with eight identified isoforms of the regulatory subunit, is encoded by three genes that undergo alternative splicing [144]. Phosphorylated Irs serves as a docking protein for the PI3K p85 subunit, and the docking leads to activation of the p110 catalytic subunit. To evaluate the role of the regulatory subunit of PI3K in the liver, mice lacking any isoform of the PI3K regulatory subunit in the liver (L-p85DKO mice) were generated by crossing the hepatocyte-specific Pik3r1 gene (producing p85α, p55α, and p50α) knockout mice with Pik3r2-knockout mice lacking the other major p85 isoform, p85β, in all the tissues [145]. L-p85DKO mice failed to show activation of PI3K and Akt by insulin stimulation, resulting in increased expressions of the gluconeogenic genes, impaired glucose tolerance, and hyperinsulinemia. In addition, defective activation of PKCλ by insulin was associated with hypolipidemia and decreased transcription of SREBP1c. In these mice, suppressed activity of PKCλ, but not of Akt, might be associated with low levels of hepatic SREBP1c expression. Similar phenotypes, including hyperinsulinemia, glucose intolerance, upregulated expressions of gluconeogenic genes, and decreased lipogenesis were observed when the hepatic PI3K activity was inhibited with an adenoviral vector encoding a dominant-negative mutant of p85 [146]. Mice with hepatic knockout of p110α also showed reduced insulin sensitivity, impaired glucose tolerance, increased gluconeogenesis, and decreased expression levels of several lipogenic genes in the fed state [147]. While the loss of p110β had no significant effect on liver lipid accumulation, hepatic TG levels in HFD-fed liver-specific p110α-knockout mice were lower than in the HFD-fed control mice [148]. Conversely, enhanced PI3K activity resulted in hypoglycemia and increased hepatic TG levels when miR-378, which can inhibit hepatic p110α expression via epigenetic modifications, was knocked out in the liver [149].

### 4.4. PDK1

PI3K phosphorylates and converts phosphatidylinositol-3,4-diphosphate (PIP2) into PIP3, which recruits the serine/threonine kinase phosphoinositide-dependent kinase 1 (PDK1) to the plasma membrane through its PH domain, and phosphorylates Thr308 on Akt [144]. Liver-specific PDK1-knockout (LPDK1KO) mice showed impaired Akt activation by insulin, lower hepatic glycogen contents, impaired suppression of gluconeogenesis, and impaired induction of lipogenic genes following refeeding [150]. It was thought that the reason for the death of these mice between 4 and 16 weeks of age due to liver failure was the use of the AFP promoter-driven Cre-recombinase. When a promoter of the albumin gene was used, LPDK1KO mice exhibited marked hyperinsulinemia, postprandial hyperglycemia, and lower expression levels of SREBP1c in the randomly fed condition, without liver damage or shortening of the life span [151].

### 4.5. Akt

Although Akt1, one of the three Akt isoforms, is important for cell survival and growth, Akt2 appears to play a more prominent role in the liver [144]. Reduced TG contents of the liver, a decrease in the expressions of lipogenic genes, and DNL were observed in both ob/ob mice and HFD-induced obese mice with loss of hepatic Akt2, although these changes were not observed under the condition of a normal chow diet [152]. Mice with hepatic deletion of Akt1 and Akt2 showed glucose intolerance, insulin resistance, and defects in the transcriptional response to feeding in the liver, including lipogenic gene expressions, all of which were normalized with concomitant liver-specific deletion of FoxO1 [153].

### 4.6. PTEN

Since PTEN dephosphorylates PIP3, inactivation of PTEN results in enhanced activation of the PI3K pathway. Hepatocyte-specific PTEN-knockout (LPTENKO) mice exhibited massive hepatomegaly and steatohepatitis with TG accumulation, accompanied by elevated lipogenesis gene expression levels and β-oxidation [154,155,156]. Whereas enhanced hepatocarcinogenesis was also observed, the LPTENKO mice showed not only improved glucose tolerance, but also improved systemic insulin sensitivity, caused by redistribution of the body fat from the fatty tissue to the liver, and/or increased hepatic FGF21 production. Based on a series of these results in genetically modified mouse models of insulin signaling (Table 1), hepatic insulin signaling via at least the IR/Irs/PI3K/Akt pathway is definitely important for the development of fatty liver.

## 5. Pathogenesis of Hepatic Insulin Resistance

While insulin signaling is known to be positively correlated with hepatic fat accumulation, hepatic insulin signaling is thought to be disturbed in NAFLD. As in the adipose tissue and skeletal muscle, proinflammatory cytokines, endoplasmic reticulum (ER) stress, and reactive oxygen species (ROS) cause JNK overactivation in hepatocytes, leading to hepatic insulin resistance, in part through inhibitory serine phosphorylation of the insulin signaling molecules Irs1 and Irs2 [157]. After three days of high-fat feeding with hepatic fat accumulation and hepatic insulin resistance in the absence of significant peripheral fat accumulation or peripheral insulin resistance, hepatic insulin resistance could be attributed to impaired insulin-stimulated Irs1 and Irs2 tyrosine phosphorylation, associated with activation of PKC-epsilon (PKCε) and JNK1 [158].

It is well known that Irs2 expression is suppressed by insulin signaling itself at the transcriptional level through the insulin response element via inactivated FoxO or activated SREBP proteins [159,160,161,162,163], suggesting that hyperinsulinemia, which is common in obesity, metabolic syndrome, T2DM, and NAFLD, could cause hepatic insulin resistance by downregulation of Irs2. In addition to this regulatory circuit, epigenetic regulation of Irs2 has also been reported recently. According to one study, decreased Irs2 transcription in obese patients with T2DM was associated with elevated miRNA let-7e-5p levels and altered Irs2 DNA methylation at the SP1- and SREBF1-binding sites [164]. Since hepatic Irs2 mRNA levels have been reported to be actually lower in NAFLD patients [165], downregulated Irs2 might be involved in the pathogenesis of hepatic insulin resistance.

In addition, a large number of studies have indicated that accumulation of DAG in the liver plays a crucial role in mediating hepatic insulin resistance [166]. In humans with obesity, it was found that intrahepatic TG, rather than the visceral adipose tissue area, was a better marker of the metabolic derangements associated with obesity [167]. The DAG content in cytoplasmic lipid droplets of the hepatocytes was found to be the best predictor of insulin resistance [168]. DAG is a lipid intermediate in TG synthesis. In the physiological state, an increase in TG is accompanied by an increase in DAG. DAG-mediated activation of PKCε induces inhibition of the insulin receptor (INSR) kinase activity due to INSR Thr1160 phosphorylation [169]. Subcellular distributions of DAG and PKCε seem important for INSR Thr1160 phosphorylation, based on the findings of studies using comparative gene identification 58 (CGI-58), which is a lipid droplet-associated protein that promotes the hydrolysis of TG by activating adipose triglyceride lipase (ATGL). Whereas mice with knockdown of CGI-58 showed hepatic steatosis as well as increased DAG content, they remained insulin sensitive and were protected from diet-induced obesity and glucose intolerance [170,171]. Knockdown of CGI-58 caused increased DAG content in hepatocyte lipid droplets and suppressed PKCε translocation to the plasma membrane, suggesting that intracellular compartmentalization of DAG is key to the interaction of DAG and PKCε [171]. Among the three known DAG stereoisomers, increased content of sn-1,2-DAG in the plasma membrane has been observed in both humans with hepatic insulin resistance and rats with hepatic insulin resistance induced by acute hepatic DGAT2 knockdown, which results in enhanced phosphorylation of INSR Thr1160 [172]. (Figure 3)

## 6. How Is Hepatic Lipogenesis Increased Even in the Presence of Hepatic Insulin Resistance?

While impaired suppression of hepatic gluconeogenesis is caused by hepatic insulin resistance, increased hepatic DNL is also observed in NAFLD. As hepatic insulin signaling positively regulates hepatic DNL, the condition in NAFLD wherein insulin fails to suppress gluconeogenesis but continues to activate DNL is referred to as “selective hepatic insulin resistance” [173]. A number of studies have attempted to identify the branch point in hepatic insulin signaling at which its effects on glucose and lipid metabolism diverge, and also other factors that induce hepatic lipogenesis, such as increased lipogenic substrate supply from other tissues, which are independent of hepatic insulin signaling (Figure 3).

### 6.1. Point of Divergence of Glucose and Lipid Metabolism in the Liver

As described above, SREBP1c is regulated at both the transcriptional level and at the level of proteolytic processing by mTORC1 [130]. Whereas inhibitors of PI3K and Akt blocked insulin-mediated increase in SREBP1c mRNA expression and suppression of gluconeogenic genes, rapamycin, an inhibitor of the mTORC1, blocked insulin-mediated induction of SREBP1c, while having no effect on insulin-mediated suppression of the gluconeogenic genes [174]. As the mTORC1 pathway senses nutrients such as amino acids through Rag GTPase, leading to the translocation of mTORC1 to the lysosomes [175], mTORC1 is considered as being one of the causes of selective hepatic insulin resistance. However, based on the findings of recent studies, activation of mTORC1 alone is not sufficient to induce DNL and development of steatosis. Stimulation of mTORC1 and SREBP1c is not sufficient to drive postprandial lipogenesis in the absence of Akt2 [176]. Akt activates mTORC1 through phosphorylation and inhibition of the TSC2 protein within the TSC1–TSC2 complex. Liver-specific deletion of TSC1 (LTsc1KO), which results in insulin-independent activation of mTORC1, protected against age- and diet-induced hepatic steatosis and led to hepatocyte-intrinsic defects in SREBP1c activation and DNL [177,178]. These phenotypes were thought to be due to decreased Akt activation through a negative feedback loop in LTsc1KO, especially impaired Akt-mediated suppression of Insig2a, a liver-specific transcript encoding the SREBP1c inhibitor INSIG2 [178]. From the results of experiments using mice lacking hepatocyte-specific Akt1, Akt2, FoxO1, and TSC1, the coincident inhibition of FoxO1 and activation of mTORC1 by insulin was reported to be both necessary and sufficient for activation of DNL [179].

### 6.2. Substrate-Driven Increase in Hepatic DNL

Since hepatic insulin resistance caused by the DAG–PKCε pathway occurs at the level of phosphorylation of the insulin receptor, it is reasonable to assume that insulin-independent DNL occurs in cases of MAFLD with nutrient oversupply. Carbohydrate-response element binding protein (ChREBP) is a transcription factor that is known to mediate the glucose responsive lipogenic pathway. ChREBP expression in the liver has been shown to be elevated in both patients with obesity [180] and NASH [181]. Transient overexpression of ChREBP in the liver results in hepatic steatosis without insulin resistance through induction of the lipogenic enzyme Scd1 [181]. Transient liver-specific inhibition of ChREBP in ob/ob mice markedly improved hepatic steatosis by decreasing DNL through suppression of the lipogenic enzymes ACC and FAS [182]. Similarly, hepatocyte-specific ChREBP-knockout mice were protected against carbohydrate-induced hepatic steatosis in spite of showing impaired glucose tolerance [183]. Thus, ChREBP seems to be a key factor for understanding the pathophysiological condition of increased DNL existing concomitantly with hepatic insulin resistance. Insulin-independent regulation of fatty acid esterification has also been reported. Using LC–MS/MS, it was found that the rate of fatty acid esterification into hepatic TG was dependent on the plasma fatty acid infusion rates, but independent of changes in the plasma insulin concentrations and hepatic insulin signaling [184]. Recently, Ter Horst et al. reported the results of clinical research in obese patients with and without NAFLD [185]. Obese patients with NAFLD not only showed impaired insulin-mediated suppression of glucose production and increased DNL after overnight fasting, but also attenuated glucose-stimulated/high-insulin lipogenesis. Fructose ingestion is also known to robustly stimulate hepatic DNL, and fructose-stimulated/low-insulin lipogenesis was thought to be intact in NFALD. Hepatic insulin signaling in liver biopsy samples collected during bariatric surgery was found to be attenuated at the level of insulin receptor phosphorylation after administration of glucose. Moreover, the expression levels of ChREBPβ (active isoform), but not ChREBPα, were found to be increased in patients with NAFLD, both after administration of glucose and in the fasting state. These data suggest that an increase in substrate-driven hepatic DNL via ChREBP may be one of the plausible mechanisms to explain increased hepatic DNL in MAFLD with hepatic insulin resistance.

### 6.3. Hepatic Zonation of Irs1

As increased hepatic DNL in obese NAFLD patients has recently been reported to be inversely correlated with hepatic and whole-body insulin sensitivity and directly correlated with the 24 h plasma glucose and insulin concentrations [186], it is still possible that enhanced hepatic insulin signaling resulting from hyperinsulinemia in cases of NAFLD directly promotes hepatic DNL. Among mouse models of diet-induced obesity (DIO) with hepatocyte-specific knockout of various genes related to insulin signaling (Table 1), LIrs2KO mice, but not LIrs1KO and LIrs1/2DKO mice, were not protected from hepatic steatosis in spite of the partial impairment of hepatic insulin signaling. We previously identified that hepatic zonation of Irs1 plays a crucial role in selective insulin resistance [143]. It is known that the hepatic periportal (PP) zone is the primary site of gluconeogenesis, whereas the hepatic perivenous (PV) zone is the primary site of lipogenesis. Among the molecules of the insulin signaling pathways, such as IR, Irs1, Irs2, Akt1, and Akt2, only the expression of Irs1 is twofold higher in the PV zone than in the PP zone. Under an HFD condition, the phosphorylation levels of Akt are decreased in the PP zone due to suppressed expression of Irs2 at the transcriptional level by the hyperinsulinemia associated with DIO. On the other hand, the phosphorylation level of Akt is increased in the PV zone, because of the intact expression levels of Irs1 in the absence of suppression by hyperinsulinemia. While insulin signaling in the PP and PV zones is impaired in both LIrs1KO and LIrs1/2DKO mice, it is impaired only in the PP zone in LIrs2KO mice. Due to the abundant expression of Irs1 in the PV zone, insulin signaling is considered to be rather enhanced in the presence of hyperinsulinemia despite the downregulation of Irs2, resulting in increased lipogenesis and development of steatosis. Wnt/β-catenin signaling is assumed to be involved in the hepatic zonation of Irs1. While the stabilized and active form of β-catenin is localized in the hepatocytes of the PV zone, a negative regulator of β-catenin, the adenomatous polyposis coli (APC) protein, is expressed in a complementary manner to inactivate β-catenin in the hepatocytes of the PP zone. Indeed, the β-catenin/TCF4 complex directly binds to the Irs1 promoter. Moreover, expression of Irs1 was upregulated in mouse primary hepatocytes stimulated with recombinant Wnt3a, which can activate canonical Wnt/β-catenin signaling [187]. In immunohistochemical staining of liver biopsies from NAFLD patients, the staining intensities of β-catenin and Irs1 seemed to be positively correlated [188]. While downregulation of Irs2 accompanied by decreased gluconeogenic gene expressions was observed in both patients with simple steatosis and NASH, expression of *FAS*, a lipogenic gene, was not decreased and was strongly correlated with Irs1 expression in NAFLD, suggesting that selective insulin resistance via downregulation of Irs2 is present in the early stages of NAFLD in humans [165]. All these data lend support to the notion that differential hepatic distribution of Irs1/Irs2 could be one of the mechanisms underlying the selective insulin resistance observed in MAFLD.

### 6.4. ER Stress

Enhanced inflammation, ROS, and ER stress observed in NAFLD directly or indirectly lead to subsequent steatosis and disease progression [7]. ER stress is caused as a response to the accumulation of unfolded proteins in the endoplasmic reticulum (ER). The unfolded protein response (UPR) gene is activated to varying degrees in the liver of patients with NAFLD [189]. ER stress has been reported to be related to the progression of NAFLD through causing dysregulated apoptosis, increased generation of ROS, and exacerbation of hepatic insulin resistance caused by IRE1α-dependent JNK activation [190]. Recently, Sasako et al. demonstrated that “ER stress response failure”, as well as enhanced ER stress, was associated with progression of insulin resistance and steatohepatitis [191]. Although the relationship between ER stress and hepatic DNL is not yet fully understood, Kim et al. identified the mechanism of ER stress-driven DNL [192]. Elevated caspase-2 expression was observed in ER-stressed mice and human with NASH, and caspase-2 ablation abolished SREBP1/2 activation in the liver of ER-stressed mice. In addition, pharmacological inhibition of caspase-2 also prevented hepatic steatosis, fibrosis, and inflammation. Activation of SREBP1/2 by increased caspase-2 activation was considered to be caused by increased cleavage of site 1 protease (S1P), leading to SCAP-independent SREBP1/2 activation in the ER. Thus, ER stress might contribute to the development of increased hepatic DNL in hepatic insulin resistance, at least in the advanced stages of NAFLD/NASH.

## 7. Conclusions

With a vast majority of studies reporting the etiological heterogeneity and pathogenesis of NAFLD, it has become clear that metabolic dysfunction is closely involved in the complex mechanism underlying the development of NAFLD, prompting a call to consider switching the term NAFLD to MAFLD. The metabolic dysfunction mentioned above consists of obesity, T2DM, hypertension, dyslipidemia, and metabolic syndrome, with insulin resistance as the common underlying pathophysiology. Imbalance between energy intake and expenditure leads to insulin resistance in various tissues and alterations of the gut microbiota, resulting in fat accumulation in the liver. Recent studies have also revealed the roles of some genetic factors involved in the development and progression of MAFLD. During the process of fat accumulation, intracellular damage, as well as hepatic insulin resistance, further potentiates inflammation, fibrosis, and carcinogenesis. Hepatic insulin resistance is aggravated by inhibition of physiological phosphorylation of the insulin receptor via the DAG–PKCε pathway and downregulation of hepatic Irs2 expression via hyperinsulinemia. As reasons for the impaired suppression of hepatic gluconeogenesis with increased hepatic DNL in the presence of hepatic insulin resistance, increased lipogenic substrate supply from other tissues, hepatic zonation of Irs1, and some other factors, including ER stress, may be important (Figure 3). Since MAFLD can be regarded as a hepatic phenotype of systemic insulin resistance, renaming NAFLD as MAFLD may enable more precise recognition and comprehension of fatty liver disease, with beneficial effects in basic research, clinical practice, and public health.

## Figures and Tables

**Table 1:** Summary of genetically modified mouse models of insulin signaling. (N/A: not applicable).

Type of Mice	Body Weight	Glucose Tolerance	Plasma Insulin	Hepatic Lipogenesis	Hepatic TG Content	Comments
LIRKO	Decreased, then catch up	Severely impaired	Increased	Decreased	Decreased in DIO, ob/ob mice	Metabolic phenotype becomes less severe with aging.
LIrs1KO	Normal	Mildly impaired (refeeding state)	Increased (re-feeding state)	Decreased (re-feeding state)	Decreased in DIO mice	N/A
LIrs2KO	Normal	Mildly impaired (fasting state)	Increased (fasting state)	Decreased (fasting state)	Similar to controls in DIO mice	N/A
LIrs1/2DKO	Normal	Impaired	Increased	Decreased	Decreased in DIO mice	N/A
LPI3K(p85)DKO	Normal	Impaired	Increased	Decreased	N/A	Small liver, liver dysfunction
LPI3K(p110α)KO	Increased, with increased fat mass	Impaired	Increased	Decreased	Decreased in DIO mice	Small liver, normal morphology
LPDK1KO	Normal	Impaired	Increased	Decreased	N/A	N/A
LAkt2KO	Normal	Normal	Normal	Normal	Decreased in DIO, ob/ob mice	N/A
LAkt1/2DKO	N/A	Impaired	Increased	Decreased	N/A	N/A
LPTENKO	Normal	Improved	Decreased	Increased	Increased	Development of HCC with aging

## Abbreviations

ACC	Acetyl-CoA carboxylase
CVD	Cardiovascular disease
CIDEA	Cell death-inducing DNA fragmentation factor alpha-like effector A
ChREBP	Carbohydrate-response element binding protein
DAG	Diacylglycerol
DIO	Diet-induced obesity
DNL	De novo lipogenesis
ER	Endoplasmic reticulum
FAS	Fatty acid synthase
FFA	Free fatty acid
FoxO1	Forkhead box O1
FSP27	Fat specific protein 27
GLUT	Glucose transporter
HBV	Hepatitis B virus
HCV	Hepatitis C virus
HCC	Hepatocellular carcinoma
HFD	High-fat diet
IKKβ	Inhibitor of nuclear factor kappa-B kinase subunit β
IL-6	Interleukin-6
INSR	Insulin receptor
Irs	Insulin receptor substrate
JNK	c-Jun N-terminal kinase
LPS	Lipopolysaccharide
NAFL	Non-alcoholic fatty liver
NAFLD	Non-alcoholic fatty liver disease
NASH	Non-alcoholic steatohepatitis
NEFA	Non-esterified fatty acid
NF-kappa	Nuclear factor-kappa B
MAFLD	Metabolic dysfunction-associated fatty liver disease
miRNA	microRNA
MOGAT1	Monoacylglycerol O-acyltransferase 1
OGTT	Oral glucose tolerance test
PDK1	Phosphoinositide-dependent kinase 1
PI3K	Phosphatidylinositol-3 kinase
PKC	Protein kinase C
PLIN2	Perilipin 2
PPAR	Peroxisome proliferator-activated receptors
PTEN	Phosphatase and tensin homolog
ROS	Reactive oxygen species
SCD1	Stearoyl-CoA desaturase-1
SOCS3	Suppressor of cytokine signaling 3
SREBP	Sterol-regulatory element binding protein
T2DM	Type 2 diabetes mellitus
TCA cycle	Tricarboxylic acid cycle
TG	Triglycerides
TGF-β	Transforming growth factor-β
TLR-4	Toll-like receptor-4
TNF-α	Tumor necrosis factor-α
TSC	Tuberous sclerosis complex
